# Thermo-alkali stable bacterial xylanase for deinking of copier paper

**DOI:** 10.1186/s43141-023-00563-0

**Published:** 2023-10-25

**Authors:** Girisha Malhotra, Shilpa S. Chapadgaonkar

**Affiliations:** 1 Manav Rachna International Institute of Research and Studies, Faridabad, Haryana India; 2 Dr. Vishwanath Karad MIT World Peace University, Kothrud, Pune, Maharashtra India

**Keywords:** Xylanase, Bio-bleaching, Bleach-boosting, Thermostable, Bio-pulping, Deinking

## Abstract

**Background:**

The bleach-boosting capability of xylanases is well-known. The use of xylanase pre-treatment before the application of chemical bleach has multiple advantages including (i) lesser use of polluting chemicals of the traditional bleaching process; (ii) less damage to the cellulosic fibers, therefore better recyclability; and (iii) better brightness of chemical bleach. The major impediment in the application is the availability of commercial enzymes that are active at the elevated temperature and pH that exist during the industrial pulping process. In the present paper, xylanase having suitability for application in deinking is reported.

**Results:**

The xylanase used showed high deinking potential. Optimal deinking was obtained at the xylanase dosing of 20U/g of the dried pulp at 60℃ for a treatment time of 1h. It could bring about a 50% reduction in the usage of chemical bleach that was applied after xylanase pre-treatment. The comparison of FTIR spectra showed changes in intensity without significant changes in the functional group signatures implying that there is negligible damage to the fiber strength in the xylanase pre-treatment process as compared to the chemical bleach process.

**Conclusion:**

The xylanase used in this study was effective in deinking paper pulp and can be used for bio-bleaching of recycled paper.

## Background

According to the statistics maintained by the United States Environmental Protection Agency (ePA. gov), each ton of recycled paper can save 17 trees, 1500L of oil, 2.3 m^3^ of landfill space, 4000 kilowatts of energy, and 26500L of water [1]. Wastepaper, therefore, is a valuable renewable raw material for the paper industry which not only reduces manufacturing costs but also reduces the use of pollutants and toxicants and saves trees. As per the statistics published by the World Economic Forum only about 68% of paper is recycled globally. This indicates that there is still an enormous potential for increasing recycling.

Traditional chemical deinking processes use strong bases, chemical bleaches, chelating agents, silicates, and surfactants. However, the use of toxic chemicals for the chemical recycling process defeats the purpose. The paper recycling process consists of defibering, deinking, and reforming. Defibering disintegrates the paper into fibers, and in the reforming process, paper is made from recycled pulp. Deinking is the critical step where the ink is removed from the paper. The chemical deinking process is not only polluting but also leads to the deterioration of fiber strength, yellowing, and wastage.

Physical pre-treatment methods are applied to the recycled pulp to aid the deinking process. Processes like sonication, microwave, and heating are often integrated with traditional methods to reduce the chemical requirement. One of the emerging sustainable alternative methods is the enzymatic pretreatment method popularly known as bio-pulping [2]. Bio-pulping exploits microorganisms or microbial enzymes as a pre-treatment for pulp deinking. The benefits of this process include a reduction in electricity consumption and an increase in output.

The enzyme xylanase has been extensively studied for bio-pulping as it is an excellent bio-bleaching agent. Bio-bleaching with xylanases has been reported to reduce the requirement of chemical bleach agents and has been shown to preserve paper quality [3]. A cocktail of enzymes, xylanases, cellulases, pectinases, and lipases has been employed for enzymatic deinking. Enzymes remove the ink from the paper pulp fiber either by breaking the chemical bonds between ink and paper or by changing the surface of the paper. After the enzymatic pre-treatment step, the free ink particles are removed by flotation or washing.

The concentration of chemical bleach required after bio-pulping varies with the type of pulp and enzymes applied [4]. Khandeparkar and Bhosle reported a reduction of up to 29% of chlorine by using bacterial xylanase [5]. Xylanase-assisted bio-bleaching of the several types of Kraft pulps was conducted by various authors with successful results in terms of bleaching, paper quality, and reduction in chemical bleach usage [6].In the present paper, we report the utility of thermo-alkali stable xylanase for the deinking of copier paper.

## Methods

The production and purification of xylanase used in the present study have been described elsewhere [7, 8]. This enzyme was thermostable and retained its activity at temperatures above 70℃ and highly alkaline conditions like pH 9. This enzyme was applied for the deinking of copier paper [9].

### Deinking of recycled print paper

An A4 size copier paper was printed with black ink using an HP laser printer. This paper was shredded and ground with distilled water. The paper pulp obtained was treated with partially purified xylanase. This was conducted by taking 5% (W/V) of pulp and treating it with varying concentrations of xylanase (10–50 U/g of pulp) of partially purified xylanase. The time for xylanase treatment was optimized by varying the treatment time between (15–90 min) keeping the enzyme dosing fixed at the optimized concentration of 20U/g for a time of 1h at 50℃ with gentle shaking. The peroxide bleaching was carried out with 10% paper pulp with 3% peroxide, 3% sodium silicate, 0.3% Diethyl triamine penta-acetic acid (DTPA), and 1.5% NaOH at 90℃ for 60 min [10]. Oven-dried pulp was grounded into powdered form in a mixer-grinder and analyzed by Fourier Transformed Infrared (FTIR) spectrometry (Devansh Testing and Research Laboratory Pvt. Ltd., Roorkee). FTIR spectra were recorded in a wave number range of 4000–500 cm^−1^ [11].

The effectiveness of xylanase pretreatment in reducing the chemical bleach was determined in a combined process where the pulp was first treated with 20U/g of xylanase for 1h and subsequently treated with 50% v/v concentration of chemical bleach and deinking keeping the rest of the process constant.

After bleaching treatment, the pulp obtained was filtered and washed 4–5 times with water and pressed between weights. A control was treated similarly to the sample. The only difference was that it was conducted without the addition of xylanase. All experiments were performed in triplicates.

## Results

### Colorimetric analysis of deinking of recycled paper

**Fig. 1:**
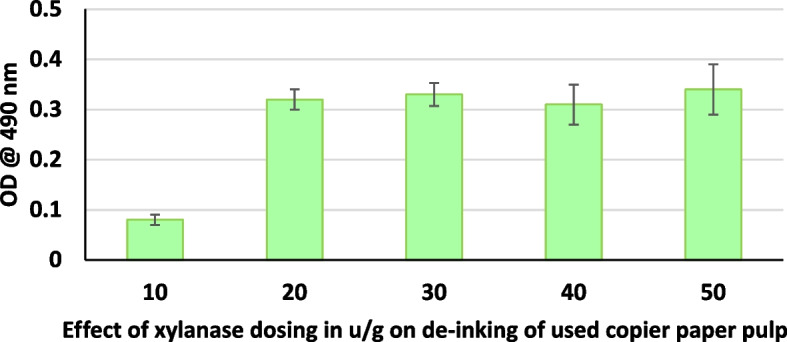
Effect of xylanase dosing in u/g on de-inking of used copier paper pulp

**Fig. 2:**
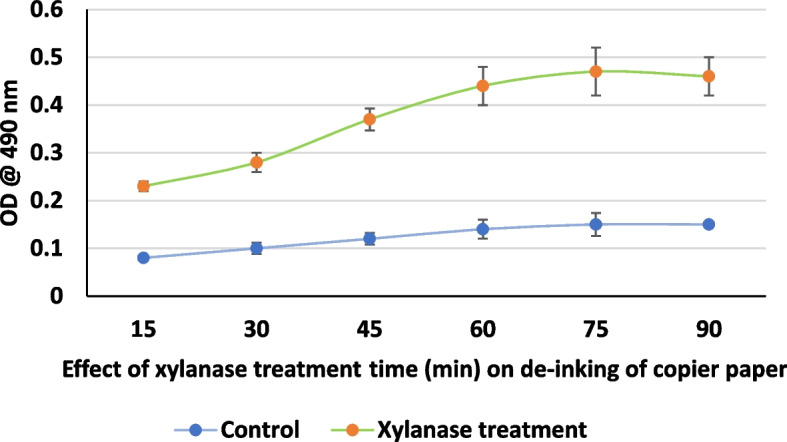
Effect of xylanase treatment time (min) on de-inking of copier paper

**Fig. 3:**
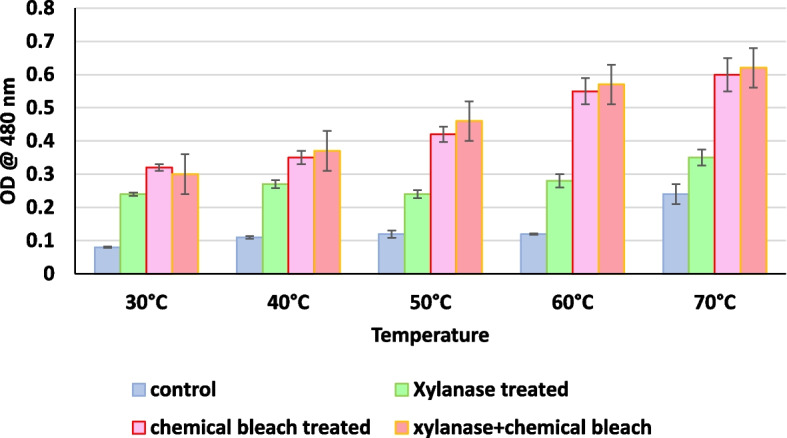
Deinking of recycled paper using xylanase pre-treatment as compared to control (treatment with water), chemical bleach, and combination of xylanase pre-treatment and chlorine bleach

After deinking the paper pulp and the supernatant was separated by centrifugation, the supernatant was analyzed colorimetrically at 490 nm for release of chromophores, from the pulp. Figure 1 illustrates the effect of enzyme dosing on deinking efficiency. An increase in enzyme dosage increases the deinking efficiency; however, an increase in enzyme dosing beyond 20U/g only marginally improves the deinking efficiency. Similarly, increasing the xylanase treatment time also enhances the deinking. A treatment time of a minimum 60min was required to give maximum deinking (Fig. 2). It can also be seen that xylanase accelerates the release of chromophores at all the temperatures evaluated. However, a temperature between 60 and 70℃ was found to be optimum (Fig. 3). Xylanase pre-treatment was able to reduce the chemical bleach requirement to 50%.

### Characterization of paper pulp using FTIR

**Fig. 4:**
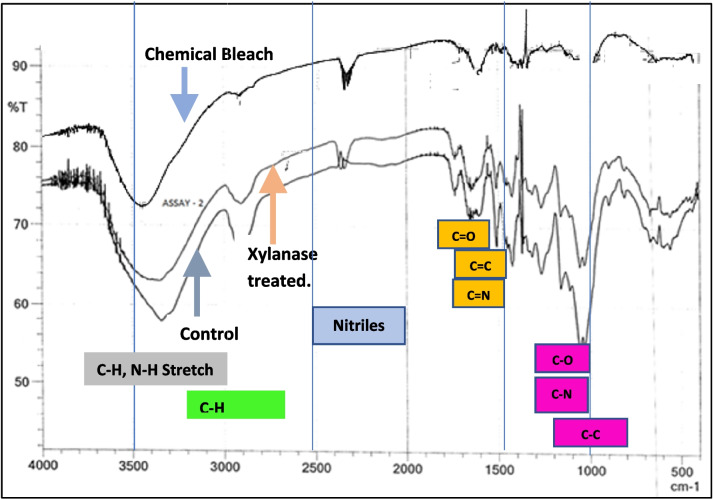
Comparison of ATR-FTIR spectra of recycled paper pulp obtained with xylanase-assisted bio-bleaching, chemical bleaching, and control (without xylanase or chemical bleach)

The paper pulp was analyzed by FTIR (Fig. 4) analysis to know the surface properties and changes in surface functionalization.

The spectra and the bond intensities obtained in different samples show significant differences in intensities (Table 1). FTIR spectra obtained with xylanase-treated pulp were compared with control (water) and chemical bleach (H_2_O_2_)-treated pulp samples. The bands around 3448–3338 cm^−1^ depict –OH stretching of H-bonding. The increase in their relative intensity after enzymatic treatments is attributed to the increase in cellulosic content of the pulp. Relative intensity around 1637–1629 cm^−1^ increased in xylanase-treated pulp attributed to the release of free carbonyl groups (C = O) due to the action of an enzyme on lignin’s aromatic ring. In control pulp, these carbonyl groups remain associated with aromatic rings resulting in less absorption. An increase in intensity at 1737 cm^−1^ in xylanase-treated pulp was assigned to C = O stretching vibration in β–C = O, COOH, and ester indicating that residual lignin was enriched in these types of functional groups. The increase in the intensity of the bands around 1162 cm^−1^ in xylanase-treated pulp indicated the degradation of syringyl groups. The treatment with chemical bleach is seen to affect some of the structural bonds in cellulose, thereby reducing the fiber strength and recyclability [3].

**Table 1:** Comparison of FTIR spectrum of the xylanase-treated paper pulp with control and chemical bleach-treated recycle paper pulp

Assignment of bonds	Mode of vibration	**Control**		**Xylanase-treated**		**Bleach-treated**	
		Peak	Intensity	Peak	Intensity	Peak	Intensity
N–O	Stretch	669.3	68.474			669.30	93.348
= CH_2_	Wagging	812.03	73.833	812.03	77.362	813.96	97.79
= CH2	Wagging	896.9	72.882	898.83	77.638	896.9	98.493
C–O	Streching	1033.85	54.673	1035.77	65.454	1049.28	93.136
C–O	Streching	1058.92	54.2	1058.92	65.158	1093.64	94.707
C–O	Streching	1112.93	62.24	1112.93	71.323	1112.93	94.525
–C–O	Streching	1161.15	62.457	1161.15	71.425	1161.15	95.214
–C–O	Streching	1267.23	64.349	1267.23	72.507	1267.23	95.931
–C–H(CH_3_)	Bending symmetric	1319.31	66.489	1317.38	74.172	1319.31	96.09
–C–H(CH_3_)	Bending symmetric	1334.74	67.052	1334.74	74.517	1338.60	95.729
–C–H(CH_2_)	Bending (scissoring)	1502.55	70.05	1502.55	76.405	1508.33	94.927
–C–H(CH_2_)	Bending (scissoring)	1510.26	67.722	1510.26	74.926	1516.05	95.889
–C = C–(cis)	Streching	1629.85	71.575	1620.21	75.767	1627.92	92.35
–C = C–(cis)	Streching	1637.56	71.111	1637.56	74.809	1635.64	91.66
–C = O(ester)	Streching	1735.93	73.971	1735.93	78.722	1734.01	95.63
OH	Streching asymetric					2360.87	89.778
C≡N	Streching	2347.37	77.541	2358.94	77.285	2341.58	90.36
–C–H(CH_2_)	Streching asymetric	2902.87	67.243	2904.8	72.737	2924.09	88.72
O–H	Streching	3338.78	57.862	3448.72	63.549	3446.79	71.257
O–H	Streching	3448.72	61.003				

## Discussion

The pursuit of suitable biocatalysts that could substitute the existing chemicals with greener and sustainable processes is never-ending. In the present study, the excellent deinking capability of thermo-alkali stable xylanase obtained from a newly isolated strain of *Bacillus licheniformis* has been reported. The parameters like the pre-treatment method, enzyme dosing, and time of treatment are crucial factors that affect the deinking efficiency [12]. It could be seen that the increase in the enzyme dosing, treatment time, and temperature increases the efficiency of deinking as has been reported by other researchers [2–5, 13].

These parameters were optimized for the xylanase in use. It was found that optimum deinking could be obtained at 20U/g xylanase treatment at 60℃ for 1h. When used in combination with chemical bleach, the chemical bleach concentration could be reduced to 50% of the original as reported by other researchers [14].

## Conclusion

The xylanase used in this study was effective in deinking paper pulp and can be used for bio-bleaching of recycled paper. The dosing of 20U/g of the partially purified xylanase in the study was found to be optimal for deinking purposes. In the tested dosing range, the treatment time of 60 min (1 h) was found to be sufficient for deinking. Xylanase deinking can be optimally performed at temperatures between 60℃ and 70℃. The FTIR spectrum results demonstrate significant differences in the pulp surface functional properties in control and xylanase-treated samples. These changes occur due to the release of hemicellulosic fraction as well as from the removal of chromophoric groups.

## Data Availability

The datasets used and/or analyzed during the current study are available from the corresponding author on reasonable request.
